# Raw and Pre-Treated Styrene Butadiene Rubber (SBR) Dust as a Partial Replacement for Natural Sand in Mortars

**DOI:** 10.3390/ma17020441

**Published:** 2024-01-17

**Authors:** Krzysztof Pikoń, Nikolina Poranek, Marcin Marczak, Beata Łaźniewska-Piekarczyk, Waldemar Ścierski

**Affiliations:** 1 Department of Technologies and Installations for Waste Management, Faculty of Energy and Environmental Engineering, The Silesian University of Technology, Konarskiego 18, 44-100 Gliwice, Poland; 2 Unirubber Sp. z o.o., Zielonka 17, 59-940 Węgliniec, Poland; 3 Doctoral School, The Silesian University of Technology, Akademicka 2A, 44-100 Gliwice, Poland; 4 Scientific and Didactic Laboratory of Nanotechnology and Material Technologies, Faculty of Mechanical Engineering, The Silesian University of Technology, 44-100 Gliwice, Poland; 5 Department of Building Engineering and Building Physics, Faculty of Civil Engineering, The Silesian University of Technology, Akademicka 5, 44-100 Gliwice, Poland; beata.lazniewska@polsl.pl

**Keywords:** SBR dust, rubber recycling, circular economy, concrete

## Abstract

The circular economy (CE) is widely known for its emphasis on reducing waste and maximizing the use of resources by reusing, recycling, and repurposing materials to create a sustainable and efficient system. The CE is based on 3R—reuse, reduce, and recycle. The aim of this article is to use styrene butadiene rubber dust (SBR) in building material, constituting secondary waste in the production of SBR, which is currently disposed of as landfill. SBR is partly intended to replace the natural raw material sand. The purpose of the final material is to use it for its light weight, insulating properties, or ability to absorb vibrations and sounds. Various shares of SBR dust in mortars were tested. Some of the mortars used SBR thermal pre-treatment at temperatures of 200, 275, and 350 °C. The strength and SEM results are presented. The best pre-treatment for SBR dust is thermal treatment at 275 °C. The maximum usage of rubber dust with thermal treatment is 60% as a sand substitute. The novel finding of this study is the possibility to use more than 30% rubber dust (as a substitute for sand) thanks to pre-treatment, whereby 30% is a common maximum ratio in mortars.

## 1. Introduction

The consistent goal of the circular economy (CE) adopted by the European Union [1,2] and the Sustainable Development Goals adopted by the United Nations [3] is to protect the natural environment and extend the life cycle of materials and products [4], especially SDG12 and SDG13. SDG12 aims to ensure sustainable consumption and production patterns worldwide. It addresses the efficient use of resources, reduction of waste and pollution, and promotes sustainable lifestyles and practices. SDG13 aims to take urgent action to combat climate change and its impacts by implementing measures to mitigate its effects, adapt to changing conditions, promote resilience in communities, and mobilize efforts to address the challenges posed by a warming planet [3,4].

The use of waste as a raw material is environmentally and economically justified. Achieving environmental goals also applies to the construction sector. Criteria for the design of nearly zero-energy buildings (NZEB) are included in the climate and energy package. The standard PN-EN 15251:2012 [5] introduces comfort criteria as thermal comfort, air quality, acoustic comfort, and visual comfort. However, [6] introduces a new criterion—vibrational comfort—as an important factor during the design of NZEB and passive buildings. Low-energy buildings include NZEB as defined by Directive 2010/31/EU and passive buildings as defined by the Passive House Institute in Darmstadt (Germany) standard. The common feature is the hermetic outer layer, which has high heat resistance and low demand for usable energy. The outer layer includes the outer walls, windows, doors, floor, and roof. The mixture presented in this article is the most suitable for thermal insulation on the floor of a building. The purpose of creating the mixture is to protect the natural resource, i.e., sand, to reduce its extraction and to use SBR dust (rubber powder), which comes from the recycling of rubber materials.

It is estimated that the global annual consumption of sand and gravel is 40–50 billion Mg. Sand is used in construction, but also in the production of glass and ceramics. Economic growth and intensively expanding megacities consume excessive amounts of this raw material. The United Nations Environment Program (UNEP) has established that there are “sand mafias” in countries including India, Cambodia, China, Kenya, Mexico, Sierra Leone, and Vietnam. Due to social and environmental abuses, in 2019, the UN recognized sand and gravel mining as an international issue. A consequence of excessive sand extraction is flooding, which causes the destruction of coastal forests, silts water, causes the death of sea fish and birds, and threatens tens of millions of people. Hence, it is important to look for sand substitutes [7,8]

In life cycle assessments (LCAs), sand plays a significant role, especially in analyzing the environmental impact of various products. Sand extraction, transportation, and use are evaluated in LCAs to understand their contributions to greenhouse gas emissions, energy consumption, and ecosystem disruption. It is considered in the LCAs of construction materials like concrete or glass, where sand is a primary component, allowing for a comprehensive understanding of a product’s environmental footprint across its entire life cycle. The LCA results highlighted the beneficial environmental footprints using substitutive building materials. “The LCA study’s results highlighted the better environmental footprints across environmental impact categories in the 10% substitution material in comparison to the use of primary materials” [9]. Using waste as raw materials also reduces production costs. In the article, SBR dust was used as a substitute for sand and the strength of the mixtures was also tested in proportions. Although it is estimated that SBR dust will show lower vibration absorption, sound absorption tests can be performed in further studies. An LCA analysis [10,11] can also be performed, which will show the size of the positive impact of SBR dust development on the environment. In connection with the existing “grey area” and the negative impact on the community, a social LCA analysis can also be performed, which will indicate social loss and the impact on individual stakeholders. Once the final mix is determined, life cycle costing can be performed to complete the total LCA [12].

A substitute for sand can be styrene butadiene rubber (SBR) dust in the construction mix. Although the only comparison of rubber fines with sand is the grain size of 0–2 mm, it can be used in insulating or vibration-absorbing materials. Rubber granulation reduces the strength of the mixture, which is why it is used, for example, in insulation from the ground. 

The tested rubber dust comes from the recycling of waste construction elements. The basic production raw material is rubber granulation used in industry, e.g., as a sports surface or playground surface.

**Figure 1:**
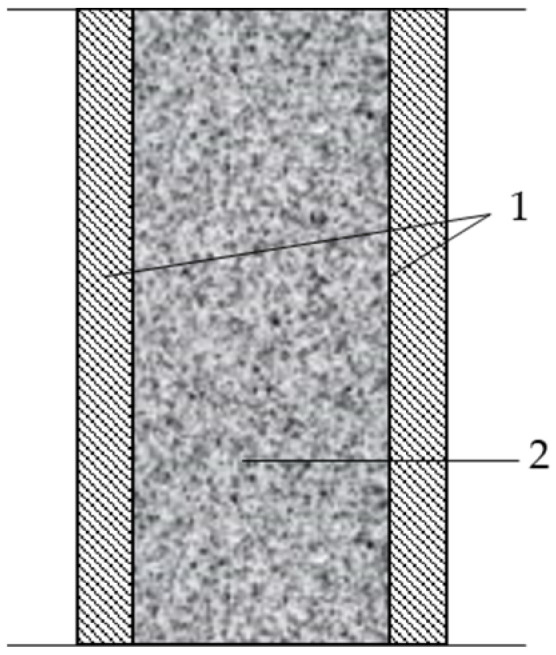
Cross-sectional diagram of a double (double-walled) partition with inhomogeneous walls. 1—walls made of heterogeneous layered partitions, 2—sound-absorbing core made of rubber granules or porous rubber.

The test results [13], and [14] showed that the addition of rubber granulate results in lower concrete density, higher impact resistance, and lower strength. The strength of the mix also depends on the granulate fraction. Concrete with granulate with larger fractions shows a greater decrease in load capacity than concrete with granulate with smaller grains. The test results showed the usefulness of rubber granulation in high-performance concretes, assuming a maximum of 15% of fine aggregate mass [15]. Other studies also indicate the possibility of modifying the mixture with SBR by adding 10% PET flakes. Studies have shown a decrease in strength with an increase in the addition of rubber in the mixture, which limits the possibility of using SBR in structural construction. However, rubber has vibration-absorbing properties, increases the frost resistance of concrete, and has insulating properties. These properties allow for a wide range of SBR applications in the construction industry. Figure 1 shows a cross divider using rubber granules. 

The sound-absorbing core is one example of the use of rubber powder, but there are many other products for which it could be used. Insulating rubber concrete is characterized by plasticity or semi-fluidity and the ability to self-level.

The properties of the set “rubber concrete” can be compared to foam concrete. The compressive strength starts from 0.5 MPa. The strength of the mix depends on the proportion of rubber powder, cement, or other materials used, e.g., recycled plastic, expanded clay, expanded polystyrene, or perlite.

In foam concrete, the shrinkage is several times greater than in ordinary concrete, which is due to the low aggregate content and relatively large amount of water in the mix. Rubber concrete avoids this problem while maintaining low density. The advantages of rubber concrete include the following:

- Quite low density (compared to traditional concrete), limiting the weight of the structure;
- High thermal insulation, making it possible to reduce the proper thermal insulation layer of partitions;
- Consistency that avoids the need to compact and level the mix;
- High biological resistance;
- Good resistance to low temperature influences;
- Low cost of production;
- The ability to shape the parameters of the material in a wide range, depending on the needs;
- Plastic or semi-liquid consistency facilitates on-site application.

The disadvantage is the lack of full knowledge about the material, including the lack of strict rules on how to design the mixture, which is the current subject of research.

**Figure 2:**
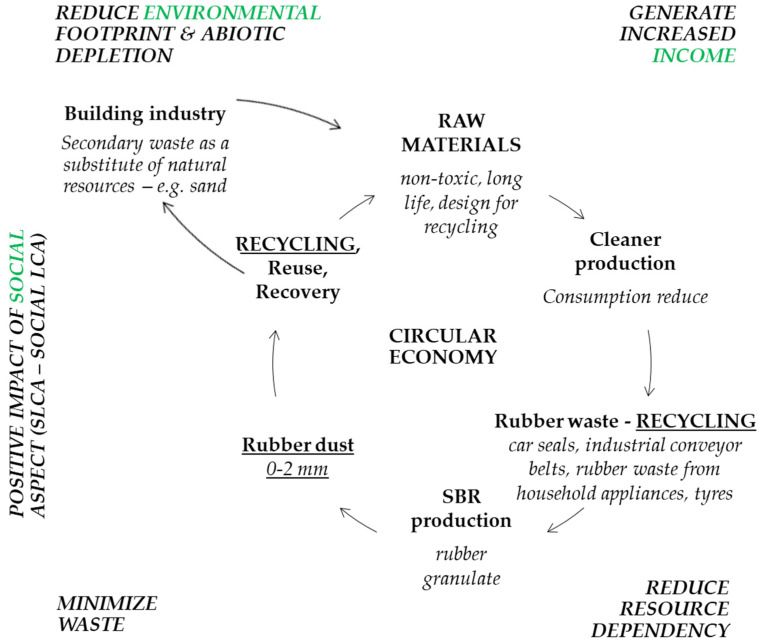
Circular economy of secondary waste during SBR production.

The novelty of the research consists in examining the possibility of using rubber powder (a by-product in the formation of rubber granulation) in the construction mix. Currently, fine coal is transported to a landfill, which does not fit into the circular economy, and the costs associated with waste storage tend to increase. One of the reasons is the fulfillment of the goal of climate neutrality by 2050, and the amount of landfilled waste by 2035 is to decrease to 10% of the stream. The use of rubber powder is an environmental and economic goal [2]. Figure 2 shows the location of rubber fines in the circular economy.

### 1.1. The Current State of Knowledge

The properties of rubber-based building materials include improved deformability, energy absorption, damping capacity, resistance to cyclic freezing and thawing, reduced water and chloride permeability, and thermal expansibility. However, the significant drawback of rubber materials compared to normal concrete is a decrease in compressive strength. This drawback can be overcome by increasing the cement content, reducing the water-to-cement ratio, and using appropriate additives. Additionally, chemical treatment (alkaline: NaOH, acidic: H_2_SO_4_), mechanical treatment (silica fume), thermal treatment (pyrolysis), UV radiation, microwave treatment, initial cement coating, or water rinsing can be applied to enhance adhesion [16,17]. These methods have resulted in strength increases of up to 56%. Rubber treatment with heat has shown a recovery of compressive strength (93%, 60%, and 47% recovery) and tensile strength (106%, 82%, and 57% recovery) when using 10%, 20%, and 30% rubber content [18].

Rubber granules have various applications. Rubber granules can be used on tennis courts or football fields. They can also be combined with polyurethane adhesive to create mats used in gyms, playgrounds, sports fields, and even as bedding for horses in stables or in cattle breeding. However, rubber dust absorbs excessive amounts of polyurethane adhesive, making it economically unviable. Rubber granules are also utilized as a component in concrete, asphalt, geopolymer composites, and flooring materials, thanks to their acoustic, vibration-absorbing, and insulating properties. It is widely known that the inclusion of rubber leads to a reduction in the strength of the mixtures [19]. Therefore, pre-treatments or additional components (fly ash, granulated blast furnace slag, etc.) are added to improve the technical properties and strength of the final product [20].

Research involving SBR dust is not widespread, but the literature suggests the use of rubber with a grain size below 1 cm in asphalt production. The dry method involves adding rubber to the mineral–asphalt mixture, while the wet method involves introducing fine-grained rubber granules (with a grain size below 1 mm) as a binder in the mineral–asphalt mixture. The rubber content in this method ranges from 10% to 20%. Various methods are employed to introduce rubber granules into asphalt in the wet method [21]. Literature also indicates the importance of pre-treatment [22,23] for achieving better properties of the material.

Rubber production is a major source of export revenue for many emerging economies, especially in the Asian region, which accounts for approximately 92% of global natural rubber production [24]. The study of Social Life Cycle Assessment (SLCA—ISO/CD 14075 under development) has revealed that the “social benefits/security” and “health and safety” of workers are the most affected elements in the social context of rubber plantations [11]. Large-scale rubber plantations in Laos have evident environmental impacts, such as the alteration of nearby stream hydrology and the decline in aquatic organisms and stream bank vegetation [25].

Global rubber production continues to rise by 1–2% annually, reaching 27.2 million Mg in 2016. The annual production volume of tires is approximately 22 million Mg, resulting in the generation of rubber waste when they reach the end of their life cycle. Considering the growing market demand for rubber products, the amount of rubber waste is also increasing. One of the leading companies in the Polish market, Unirubber, produces 1200 Mg of rubber waste annually, incurring a storage cost of 500–600 PLN per Mg. Proper utilization of this waste could save the company 660,000 PLN per year (excluding potential environmental incentives) or even eliminate waste disposal fees [26].

### 1.2. Preliminary SBR Dust Test Results

The addition of rubber dust as a substitute for aggregate in mortar negatively affects its strength. The higher the rubber content, the greater the need for modification of the mixture. The inclusion of raw rubber dust in the mortar exceeding 30% of the aggregate mass prevented proper binding of the mortar within the required timeframe [13].

The parameter examined so far is an apparent change that occurs in the state of water saturation with different raw rubber content. The results are a large release of product that comes with additional SBR dust additives. In the case of 60% content, the apparent decrease is 15% when this material is used in places where low density has an impact on relieving the structure or element.

The absorbability of mortars with the addition of SBR dust was also tested, which remains approximately constant at approximately 10% with the SBR dust content up to 30% and is comparable to the absorbability of mortars without SBR dust, which is 9.5%. After exceeding 30% of rubber content, the absorbability of mortars increases significantly [10].

Preliminary SBR dust tests recommended to use SBR dust up to a maximum of 10% of cement (which is mainly dictated by low strength). The research carried out in this article is aimed at increasing the share of SBR dust in cement mortars by using pre-treatment, such as pyrolysis.

## 2. Materials

The material subjected to granulation is obtained from production plants dealing with the production of products from the black SBR rubber mixture. The recovered material consists of rubber elements from the household appliance industry (gaskets from devices), from the industrial sector (belt conveyors, so-called rubber screeds from vulcanization works), and the automotive industry. SBR rubber waste, due to the source of origin, can be classified as post-production, i.e., originating from primary production, or secondary or post-consumer. Fully cured SBR dust, free of any metal impurities, plastic inclusions, or cord, is processed in two stages: pre-treatment and proper granulation. In the first stage, residual impurities in the rubber material are eliminated by initial grinding, removal of metal inclusions using electromagnetic sensors, granulation into a coarse fraction (approx. 30 mm), re-elimination of metal, and storage of the material prepared for final granulation. During the actual granulation, the pre-crushed material goes to the actual granulator, equipped with a sieve with the appropriate mesh size, in which the final granulation of the SBR material takes place. The granulate goes to the oscillating screen, thanks to which, in addition to the actual granulate (the final product), a side fraction from the granulation process is created—rubber fines. The bulk density of SBR rubber fines is approx. 480 kg/m^3^, with a grain size of up to 0.8 mm.

**Figure 3:**
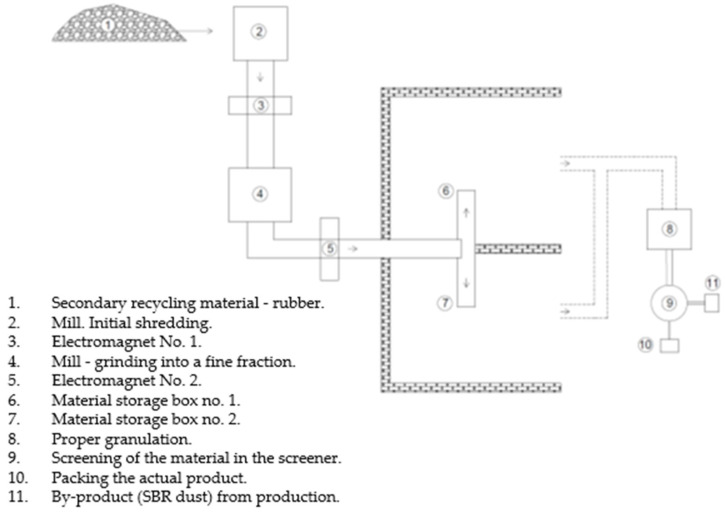
Scheme of recycling of rubber materials and indication of the place where the rubber powder is created.

Rubber waste, from which both the final product (granules for the foundation of sports surfaces and playgrounds) and post-production fines are made, is subject to the attestation procedure conducted by the National Institute of Hygiene (PZH). The condition for issuing the PZH certificate is obtaining acceptance, based on normative provisions, test results in specialized research laboratories, and a positive organoleptic assessment of the material by the PZH. The research concerns the assessment of the leachability of SBR granules using the so-called water extract, determination of the content of dissolved organic carbon (DOC), and the amount of polycyclic aromatic hydrocarbons (PAH) using the REACH method. Figure 3 shows a scheme of SBR and SBR dust production.

**Figure 4:**
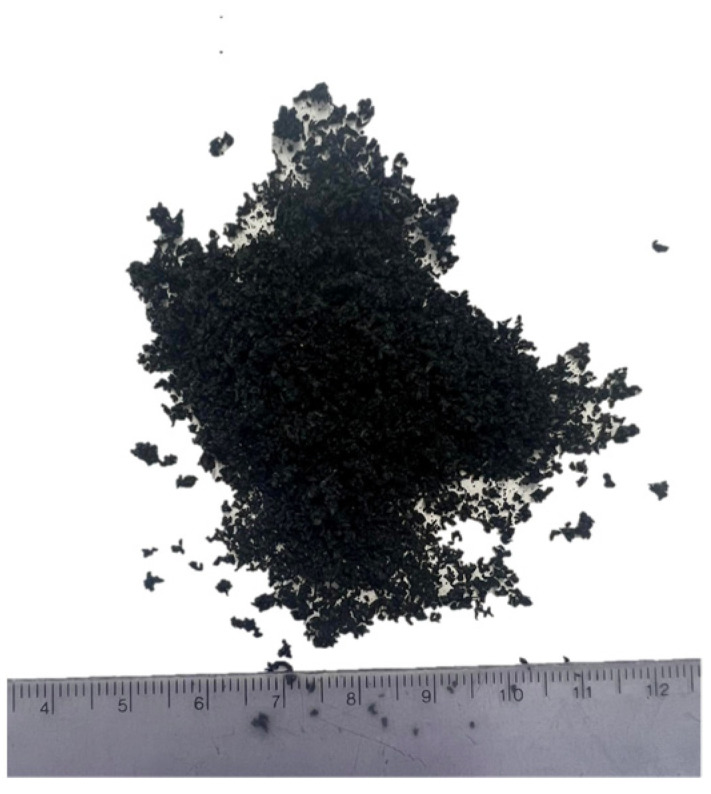
SBR dust.

The sample was a small spherical rubber granulate; the diameter of a single grain did not exceed 0.2 mm. The grain color was black, the sample had a slight smell of rubber and did not leave permanent dirt after contact with the skin. Figure 4 shows a sample of SBR dust.

Sand is a fine, natural aggregate, which in a standard mortar constitutes the majority of the mass share—1350 g. Fine aggregate was used in accordance with the PN-EN 196 standard [27].

CEM 52.5 R is a Portland cement with very fast growth dynamics of early strength. The composition of CEM 52.5 R complies with the PN-EN 197-1 standard [28]. It is usually used for the production of large-format prefabricated elements and prestressed concrete materials. It is characterized by a very high heat of hydration, which translates into shorter production times. Epoxy and polyurethane adhesives were also used for the mixes [29].

Polyurethane glue is often used in the production of rubber materials, but it is also used in construction. A feature of polyurethane glue is resistance to moisture and high flexibility. It does not change its properties under the influence of water [30].

Epoxy glue is used in construction for sealing. The feature of the epoxy adhesive is fast hardening, the possibility of using it outside and inside, thermal resistance, and activity above 8 °C [31].

Superplasticizer (SP) is a type of admixture that allows you to reduce the amount of mixing water or improve the consistency of the mixture and increase the fluidity of the mixture without changing the w/c ratio. This has a positive effect on the strength increase, which is particularly important when using SBR dust [4].

## 3. Methods

Raw SBR dust was examined under a scanning electron microscope (SEM). The analysis of the SBR surface determined the particle size of the tested material. The SEM test consists of taking a high-resolution image at high magnification, which allows the determination of the structure and topography of the material. The examination was performed using a Supra35 Zeiss with EDS detectors and WDS-EDAX from EDAX Company (Mahwah, NJ, USA) [32]. The measurement was based on the measurement of the characteristic X-ray energy spectrum.

**Figure 5:**
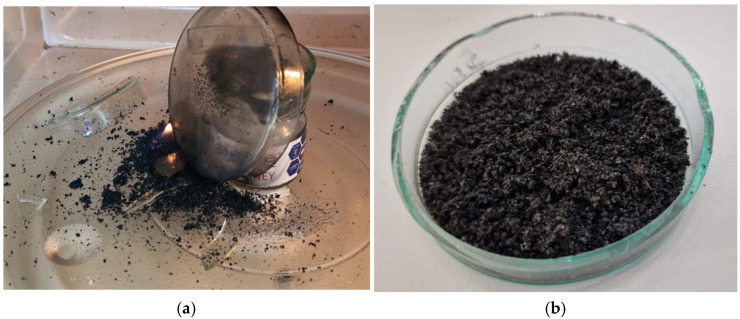
Microwave pre-treatment: (**a**) Quick and violent reaction. Less than 1 min. (**b**) Rubber dust after microwave treatment, 45 s.

In order to pre-treat the SBR dust, microwave treatment was performed, but a quick and violent response was observed, proving the low energy consumption during this process. However, the treatment needs to be refined and improved by preparing a suitable chamber or degassing device to avoid explosions and spontaneous combustion. Figure 5a shows the quick and violent reaction, causing broken glass, and Figure 5b shows rubber dust after pre-treatment (45 s).

Due to the fast reaction, and problems that could arise on a larger scale, another pre-treatment method was tested—pyrolysis was performed before adding SBR dust to the mortars. Pyrolysis is an anaerobic process usually carried out at a pressure of 0.2 MPa at a temperature of 400–1000 °C. The purpose of pyrolysis is the disintegration of particles of chemical compounds. In the thermal treatment of rubber, it was decided to use atmospheric pressure and temperatures up to 350 °C because the goal was to surface treat the rubber to increase the strength of the target compound, not to completely degrade the material. The literature presents various references discussing the rubber preparation before its use in the compound, e.g., thermal and microwave treatment, and mechanical and chemical treatment as alkaline pre-treatment. It was decided to perform preliminary pyrolysis and perform tests with the addition of glue, due to the simplest implementation of this technology in the production plant [22].

**Figure 6:**
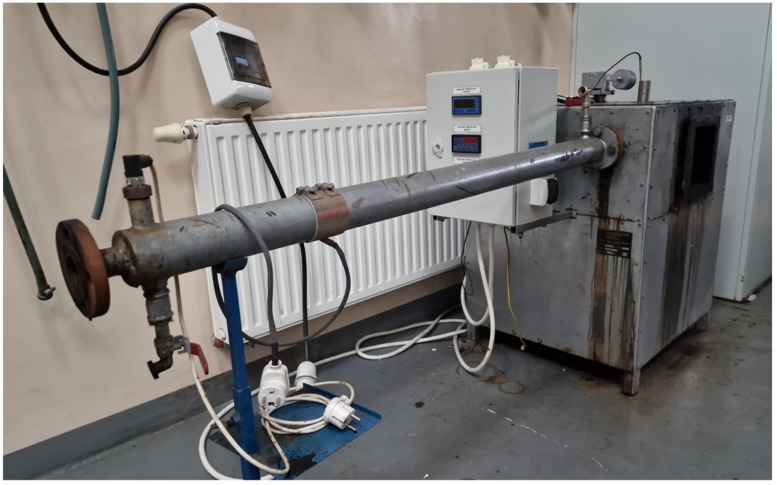
The chamber in which the thermal treatment of rubber was carried out.

In the first stage of the research, attempts were made to determine the content of volatile matter at the following temperatures: 150 °C, 200 °C, 250 °C, 275 °C, 300 °C, 350 °C, 400 °C, and 500 °C. These tests were carried out on a small scale (sample weight approx. 1 g) in order to select three characteristic sample preparation temperatures for testing their effect as an addition to cement mortars on their strength properties. After the analysis, it was decided to prepare the samples at temperatures of 200 °C, 275 °C, and 350 °C. The volatile matter content at selected temperatures is shown in Table 1. Figure 6 shows the heat treatment chamber.

The preparation of the samples consisted of heating them without access to oxygen to the assumed temperature in the reaction chamber, keeping them at this temperature until a constant weight of the samples was obtained, and cooling them down to ambient temperature.

Samples obtained at temperatures of 200 °C and 275 °C showed similar properties—the black color, a perceptible smell of rubber, and the material did not leave permanent dirt after contact with the skin.

The sample obtained at 350 °C had different properties—gray in color with no perceptible smell of rubber, the material left permanent dirt after contact with the skin. The volatile matter content was tested using Formula (1):

(1) $$V=\frac{\Delta m}{m}\cdot 100-mc\;[\%]$$

Δ*m*—loss of sample mass in the degassing process, g;
*m*—mass of the sample before degassing, g;
*mc*—moisture content, %.

In the last stage of the research, the mortars were made in accordance with the PN-EN 480-1 standard [33]. Mortars, in the form of bars with dimensions of 4 × 4 × 16 cm, were put into water for 7 days. The measurement was performed on an automatic press on which bending and compressive strength were measured. The aim of the research was to check the effect of thermal treatment and additives on the mixtures. The tests were also performed after 7 days due to the use of CEM 52.5 R.

Table 1, Table 2, Table 3, Table 4, Table 5, Table 6 and Table 7 show mortar composition with raw SBR dust, SBR dust pre-treatment with 200, 275, and 350 °C, and addition with epoxy and polyurethane glue. Sand was replaced by SBR dust in various percentages, from 10% to 60%. As the SBR dust content increases, the cement mortar becomes less brittle because the ratio of tensile strength to compressive strength increases. 

Table 1 introduces 3A, 3B, and 3C mortars with epoxy glue. The specific properties of epoxy resins allow these polymers to be classified as the best adhesive materials. The superiority of epoxy adhesives is determined by, among others, their excellent adhesion to the joined surfaces and very good chemical resistance. Table 3 introduces mortars with polyurethane glue.

Rubber surfaces (e.g., sports surfaces, playgrounds, livestock bedding) are made of rubber granules bonded with polyurethane adhesive. Polyurethane adhesives are flexible and versatile bonding materials characterized by a range of properties such as elasticity, adhesion, chemical resistance, water resistance, high mechanical strength, acoustic insulation, and resistance to aging. Despite the valuable properties of adhesives and the use of polyurethane adhesive in various surfaces, in the given proportions, they did not significantly affect the durability of the materials. However, 3C mortar with epoxy glue has higher strength than mortar with the same ingredients but without epoxy glue.

The glues could positively impact the aging properties of the material, thereby extending the life cycle of the waste. To confirm these assumptions, aging tests need to be conducted.

After testing the impact of glues on mortars, which did not give significant effects, and SBR dust, in order to compare the impact of the use of SBR dust, tests with thermal treatment T200 °C were performed. Mortars were made with 20, 40, and 60% mass fraction of rubber in relation to sand. The strength improved as shown in the summary chart and the compositions are shown in Table 4.

The strength results for T275 °C were the best among all testing temperatures. The tests were also carried out for 20, 40, and 60% mass fraction of rubber in relation to sand, as presented in Table 5.

The temperature of T350 °C was too high and adversely affected the properties of SBR dust grains. Mortars with 20% and 60% SBR dust content did not bind. High hygroscopicity also occurred, causing the mortars to crumble and become heavily soiled. The possibility of processing SBR dust at a temperature of 350 °C should be completely excluded.

Based on the tests from the first cycle, test compositions for cycle no. 2 were prepared. They are presented in the Results section.

## 4. Results

**Figure 7:**
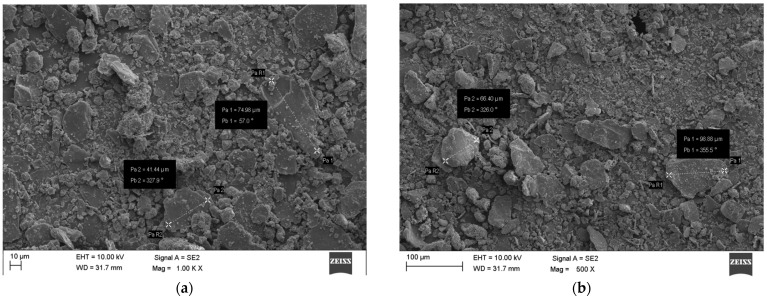
Raw SBR dust SEM. (**a**) 10 μ view of SBR dust sample; (**b**) 100 μ view of SBR dust sample.

Figure 7a,b shows SEM photos of raw SBR dust. SEM indicates a heterogeneous mixture. The grains are of different sizes with sharp, jagged edges. Most of the grains are oval in shape, but there are oblong grains. The surface of the SBR dust resembles SBR subjected to the abrasion process. Ribs with angular and prominent ridges are visible. Prolonged abrasion produces folding and cavities on the surface, but in this case, the folding is not visible. There are also no visible cracks typical of abrasion.

Table 6 shows the volatile matter of SBR dust after heat treatment at selected temperatures of 200, 275, and 350 °C.

The volatiles matter does not increase linearly. At 200 °C, it is only 0.31% and at 275 °C, it is 1.87%. The beginning of decomposition is above 275 °C and below 350 °C. Hazardous substances are produced during decomposition; therefore, based on volatile matter testing, it is recommended to use rubber after processing at 200 °C and 275 °C. Additionally, SBR dust grains heat treated at 350 °C were not elastic, which reduced the properties of the rubber. This sample was also highly hygroscopic, which had a negative impact on the mortars.

Based on the above compositions and results, the decision was made to optimize the compositions for the temperature T200 °C due to the lower energy consumption of the thermal processing and simultaneous improvement of strength. Based on the results of the 3C mortar, 6 g of epoxy glue was added to the mortars, in which the highest strength was recorded among those studied. (Mortars with SBR without thermal treatment were investigated with and without epoxy glue.)

Research was also conducted with an increased non-standard amount of cement to examine its impact on strength. The focus was primarily on temperatures of 200 and 275 °C, as well as the addition of epoxy glue. One test was performed using SBR dust at T350 °C (60% SBR dust). The test bound; however, the strength was very low, further confirming the exclusion of such a high temperature in the SBR dust preparation process.

Table 8 shows the mortar composition SBR dust 200, 275, 350 °C, epoxy glue and increased volume of cement slurry from second cycle of tests.

**Figure 8:**
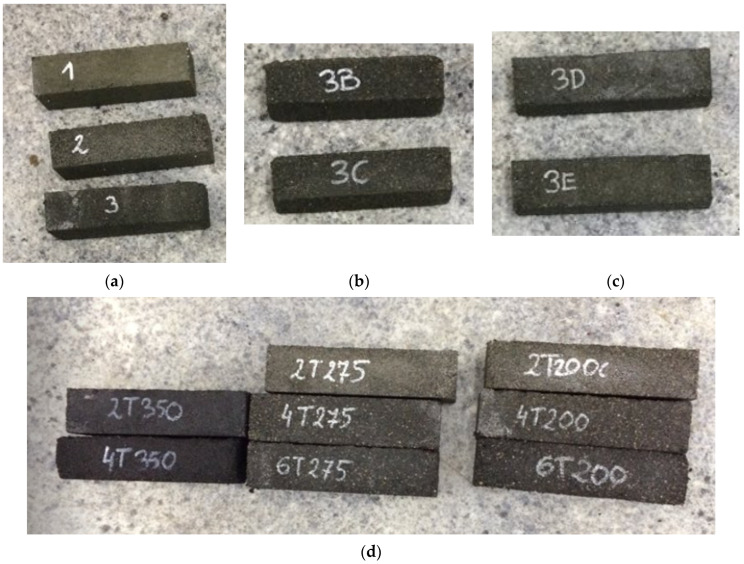
Bound samples. (**a**) Mortars with raw SBR dust (10%, 20%, and 30%). (**b**) Mortars with raw SBR dust and epoxy glue, 30%. (**c**) Mortars with raw SBR dust and polyurethane glue, 30%. (**d**) Pre-treatment SBR dust mortars at temperatures 2T200 °C (20% SBR dust to sand), 4T200 °C (40%), 6T200 °C (60%), 2T275 °C (20%), 4T275 °C (40%), 6T275 °C (60%), 2T350 °C (20%), and 4T350 °C (40%).

All tests in the second research cycle bound. However, to determine the rationale for increasing the proportion of energy-absorbing cement in mortars, it would be necessary to conduct life cycle assessments for both the carbon footprint and abiotic depletion categories. At this stage of the research, the most justified choice would be the 2T275 sample, which involves a 20% mass substitution of sand after treatment at 275 °C. The bound samples from the first series are presented in Figure 8a–c, while the bound samples from the second series are shown in Figure 8d.

**Figure 9:**
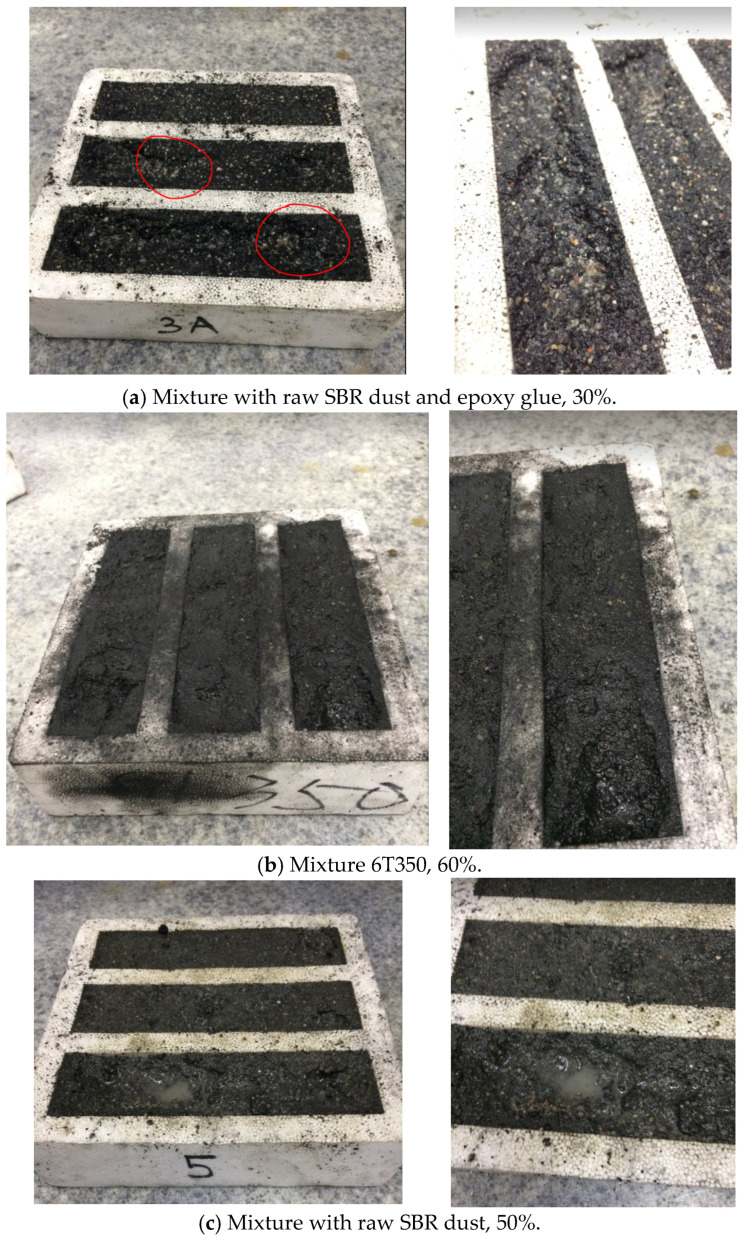
Unbound mixtures.

This research shows that the correct bonding of the mixture is affected by the selection of the optimal temperature up to 275 °C (tests carried out: 200 °C and 275 °C) and optimal selection of the glue, up to 11.5 g per mortar (where too much of it causes the mixture to not bind), and the appropriate selection of SBR dust content depending on the type of SBR dust (raw SBR dust or heat-treated). Raw SBR dust could be used up to 30%, and thermal-treated SBR dust could be used up to 60% as a sand substitute. The right amount of plasticizer (the more rubber, the more plasticizer) matters too. Figure 9 shows samples that did not bind.

Research shows that the following have a negative impact on mixtures:

- Too high rubber processing temperature (350 °C);
- Too much epoxy glue (22.5 g per mortar);
- Too high amount of raw rubber in the mixture (above 30%).

**Figure 10:**
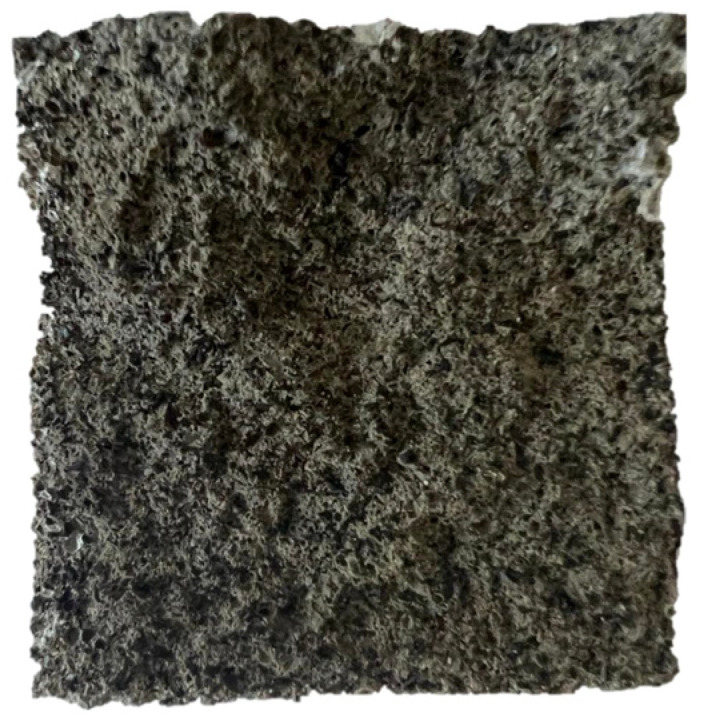
Cross-section matrix of the mortar with SBR dust.

Figure 10 shows a cross-section matrix of a compound with rubber. The compound has a dark color which is due to the black color of the rubber.

**Figure 11:**
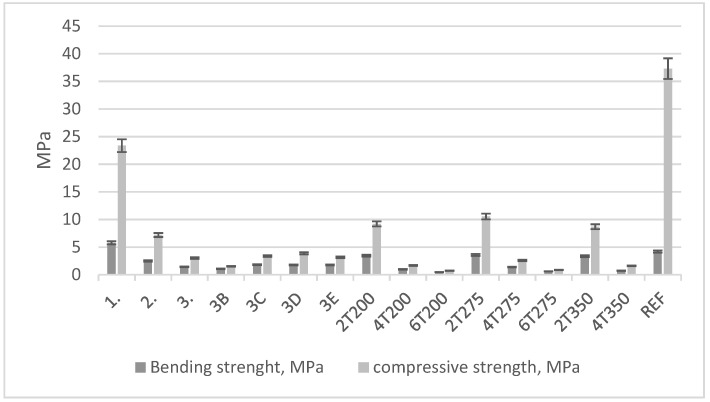
Bending and compressive strength of 1st series of research.

**Figure 12:**
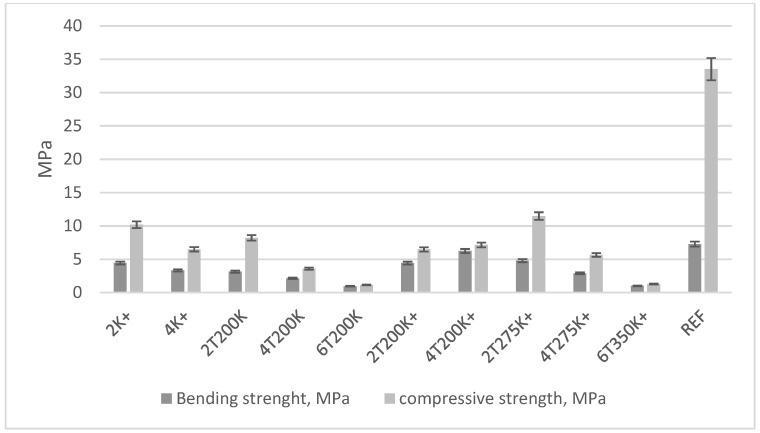
Bending and compressive strength of 2nd cycle of research.

Bending and compressive strength results are shown in Figure 11 and Figure 12. Figure 11 shows the first series of research, Figure 12 shows the second series of research.

The sample with the increased cement content, 2T275K+, exhibits the highest compressive strength. Meanwhile, the sample with the highest bending strength is 4T200K+ (with an increased cement content and 40% SBR dust by mass relative to sand after thermal treatment at 200 °C). Despite the best results for samples with increased cement content, the authors point to the optimal blends being 2T200 or 2T275, i.e., samples with SBR dust after thermal treatment at 200 and 275 °C, with a 20% mass substitution of sand.

## 5. Discussion and Conclusions

The search for substitutes for natural resources is an integral element of the circular economy and SDGs, especially goal 12 (responsible consumption and production), and 13 (climate action) [34]. Sand is a raw material widely used in industry, which is why deposits are constantly being mined. It is a critical natural resource used in construction, manufacturing, and even technology production. Its abundance might seem infinite, but certain types of sand used in specific industries are becoming scarce due to excessive extraction, leading to environmental concerns and the need for sustainable sand mining practices.

The recycling of waste extends the life cycle of materials and substances. In life cycle assessments, abiotic depletion refers to the reduction in non-living resources, such as minerals or metals, over a product’s life cycle. Sand, an essential component in various industries, particularly construction, can contribute to abiotic depletion when extracted in excessive amounts without adequate replenishment.

Sand extraction for concrete production, glass manufacturing, and other industries can deplete natural sand reserves. LCAs evaluate this depletion by quantifying the amount of sand used in a product’s life cycle and assessing its impact on natural resources. This helps in understanding the sustainability of sand usage and encourages the development of alternatives or more sustainable practices to mitigate its depletion [35].

The application of rubber in concrete mixtures can improve the concrete’s flexibility, impact resistance, and reduce its weight. These properties are often used in road surfaces to create quieter, more durable, and skid-resistant pavements. Rubber also effectively reduces noise pollution in buildings or highways. It absorbs sound vibrations, making it suitable for sound barriers or structures in noise-sensitive areas. Rubber in concrete can improve its insulating properties, reducing heat transfer. This application is useful in construction for buildings aiming for better energy efficiency. Rubber additives can enhance the durability and toughness of concrete, making it more resistant to cracking, especially in environments prone to vibrations or impacts.

Rubber granulate is used as a concrete element, but the problem is with SBR dust (secondary waste from rubber granulate production), which has, among other things, a smaller grain. SBR dust still has similar properties to rubber but is currently disposed of as landfill.

With an increase in the SBR dust content, the cement mortar becomes less brittle because the ratio of tensile strength to compressive strength increases. The addition of SBR dust results in a decrease in the apparent density of the mortar. The absorbency of the mortars remains relatively constant up to a rubber content of around 30%.

The addition of SBR dust as an equivalent of aggregate in the mortar negatively affects the strength of the mortar. The higher the rubber addition, the greater the need to modify the compound. The proportion of raw rubber in the mortar greater than 30% of the mass of the aggregate prevented the mortar from binding in a timely manner.

Thermal treatment makes it possible to increase the share of rubber from 30% to 60% in relation to the mass of the aggregate. Thermal treatment also has a positive effect on the mechanical strength of the mortar. The most optimal temperature is 275 °C. It is not recommended to use rubber roasting at T350 °C due to too-high hygroscopicity and the problem of mortar not binding, with no increase in its strength compared to mortars with rubber roasted at 275 °C, and also soiling of contact surfaces by the mortar.

The addition of epoxy and polyurethane glue to the raw rubber did not contribute significantly to the improvement of the strength properties of the mortar; however, the epoxy glue improved the strength, which may simultaneously improve the concrete aging.

The obtained research results confirm the limited applicability of SBR dust in concrete structural elements due to a significant decrease in compressive and tensile strength of the mortar. Nevertheless, after thermal treatment, the proportion of SBR dust can be increased, with a recommended limit of 20%. Mortars with the addition of SBR dust exhibit characteristics that make them suitable for use in non-structural elements. They reduce the specific weight and improve thermal insulation due to the rubber’s properties.

## Figures and Tables

**Table 1:** Mortars composition with SBR dust and epoxy glue—1st cycle.

Sample Number	SBR Dust, g	Sand, g	SP, g	Water, g	Cement, g	Epoxy Glue, g	w/c
1	135	1215	5	180	450		0.4
2	270	1080	5	180	450		0.4
3	405	945	5	180	450		0.4
3A	405	945	5	180	450	22.5	0.4
3B	405	945	5	180	450	11.5	0.4
3C	405	945	6	180	450	6	0.4
4	540	810	7.6	180	450		0.4
5	675	675	7.6	180	450		0.4
6	810	540	7.8	180	450		0.4
REF	0	1350	7.1	180	450		0.4

**Table 2:** Mortars composition with SBR and polyurethane glue—1st cycle.

Sample Number	SBR Dust, g	Sand, g	SP, g	Water, g	Cement, g	Polyurethane Glue, g	w/c
3D	405	945	5	180	450	22.5	0.4
3E	405	945	5	180	450	11.4	0.4

**Table 3:** Mortar composition with SBR dust T200 °C—1st cycle.

Sample Number	T200 °C SBR Dust, g	Sand, g	SP, g	Water, g	Cement, g	w/c
2T200	270	1080	5	180	450	0.4
4T200	540	810	9.5	180	450	0.4
6T200	810	540	7.8	180	450	0.53

**Table 4:** Mortar composition with SBR dust T275 °C—1st cycle.

Sample Number	T275 °C SBR Dust, g	Sand, g	SP, g	Water, g	Cement, g	w/c
2T275	270	1080	5	180	450	0.4
4T275	540	810	9.5	180	450	0.4
6T275	810	540	7.8	238	450	0.53

**Table 5:** Mortar composition with SBR dust T350 °C—1st cycle.

Sample Number	T350 °C SBR Dust, g	Sand, g	SP, g	Water, g	Cement, g	w/c
2T350	270	1080	5	180	450	0.4
4T350	540	810	4.8	180	450	0.4
6T350	810	540	7.5	180	450	0.4

**Table 6:** Volatile matter content at selected temperatures.

Temperature	200 °C	275 °C	350 °C
Volatile matter	0.31%	1.87%	14.35%

**Table 7:** Mortar composition with SBR dust T200 °C and epoxy glue—2nd cycle.

Sample Number	T200 °C SBR, g	Sand, g	SP, g	Water, g	Cement, g	Epoxy Glue, g	w/c
2T200K	270	1080	5	180	450	6	0.4
4T200K	540	810	9.5	180	450	6	0.4
6T200K	810	540	10.3	238	450	6	0.53

**Table 8:** Mortar composition with SBR dust 200, 275, 350 °C, epoxy glue and increased volume of cement slurry—2nd cycle.

Sample Number (Temperature)	SBR Dust, g	Sand, g	SP, g	Water, g	Cement, g	Epoxy Glue, g	w/c
2T200K+ (200 °C)	270	1080	10.1	252	630	8.4	0.4
4T200K+ (200 °C)	540	810	17.1	252	630	8.4	0.4
6T200K+ (200 °C)	810	540	21	252	630	8.4	0.4
2T275K+ (275 °C)	270	1080	15	252	630	8.4	0.4
4T275K+ (275 °C)	540	810	17.1	252	630	8.4	0.4
6T275K+ (275 °C)	810	540	19	252	630	8.4	0.4
6T350K+ (350 °C)	810	540	19	252	630	8.4	0.4

## Data Availability

Data are contained within the article.
